# Recognition and localization of ratoon rice rolled stubble rows based on monocular vision and model fusion

**DOI:** 10.3389/fpls.2025.1533206

**Published:** 2025-01-31

**Authors:** Yuanrui Li, Liping Xiao, Zhaopeng Liu, Muhua Liu, Peng Fang, Xiongfei Chen, Jiajia Yu, Jinlong Lin, Jinping Cai

**Affiliations:** ^1^ College of Engineering, Jiangxi Agricultural University, Nanchang, China; ^2^ Jiangxi Key Laboratory of Modern Agricultural Equipment, Nanchang, China

**Keywords:** ratoon rice, model fusion, depth prediction, deep learning, monocular vision

## Abstract

**Introduction:**

Ratoon rice, as a high-efficiency rice cultivation mode, is widely applied around the world. Mechanical righting of rolled rice stubble can significantly improve yield in regeneration season, but lack of automation has become an important factor restricting its further promotion.

**Methods:**

In order to realize automatic navigation of the righting machine, a method of fusing an instance segmentation model and a monocular depth prediction model was used to realize monocular localization of the rolled rice stubble rows in this study.

**Results:**

To achieve monocular depth prediction, a depth estimation model was trained on training set we made, and absolute relative error of trained model on validation set was only 7.2%. To address the problem of degradation of model's performance when migrated to other monocular cameras, based on the law of the input image’s influence on model's output results, two optimization methods of adjusting inputs and outputs were used that decreased the absolute relative error from 91.9% to 8.8%. After that, we carried out model fusion experiments, which showed that CD (chamfer distance) between predicted 3D coordinates of navigation points obtained by fusing the results of the two models and labels was only 0.0990. The CD between predicted point cloud of rolled rice stubble rows and label was only 0.0174.

## 1 Introduction

Ratoon rice is a rice cultivation method that is planted once and harvested twice (Firouzi et al., 2018), which has the advantages of short fertility, high yield, low cost and sustainable economic benefits compared to ordinary rice (Yang et al., 2024). Ratoon rice is grown in many parts of the world, mainly in East and South Asia, some countries in Africa, southern United States and Latin America (Pasaribu et al., 2018). However, existing harvesters often cause a large area of rolling damage when harvesting the first season of ratoon rice, leading to a decline in the yield of the regeneration season, which seriously affects yield and restricts further promotion of planting area (Xiao, 2018). To solve this problem, our research team developed a rolled rice stubble righting machine, which was shown to significantly increase the yield of ratoon rice (Chen et al., 2022; Chen et al., 2023). The righting machine is mounted on the back of a paddy field vehicle, and the driver needs to concentrate highly on observing the relative positions of the back of rolled stubble row and righting machine, so the driver is easily fatigued, and due to the rolled stubble row is very irregular, the righting accuracy needs to be improved. In order to solve the above problems, the righting machine needs to realize the automatic row alignment, and obtaining spatial position of rolled stubble row is a prerequisite.

Currently, Global Navigation Satellite System (GNSS), Light Detection and Ranging (LIDAR), and machine vision are the commonly used sensing methods for obtaining navigation and position information in agriculture, GNSS can only provide absolute position information, which is suitable for use under the condition of fixed position of crop rows (Bonadies and Gadsden, 2019). LIDAR can obtain the relative distances of the objects in a certain area, but it has the problems of sparse point cloud data, and it is sensitive to rain, fog, dust, etc (Zhang et al., 2022). Machine vision detects the position of objects by acquiring its color, texture, shape, and other features through vision sensors (Kim et al., 2021), which is less costly than the previous two methods, and its robustness strengthens with algorithmic enhancement, and is widely used in field navigation operations, such as weeding, tilling, spraying, etc (Zhang et al., 2024).

In crop row-based machine vision navigation applications, the common method is to first obtain the position of the navigation reference target on the image through image processing algorithms, the commonly used algorithms can be classified into image processing algorithms based on traditional image processing and based on deep learning, the deep learning model benefits from its powerful information extraction ability, even in complex environments, can also obtain high recognition accuracy, such as semantic segmentation model and instance segmentation models and so on (O’Mahony et al., 2020). Then the navigation deviation is determined based on the position of the recognized target and the declination of the forward direction (Yuan et al., 2024; Kong et al., 2025). Although this method is straightforward, it cannot acquire the metric relative distance of the navigation target with regard to the working machine, in order to solve this problem, some researchers have used multi-sensor fusion to acquire distance information while acquiring the RGB image, such as binocular camera (fusion of two monocular cameras) (Li et al., 2022), RGB-D camera (RGB camera fusion of depth sensor) (Silva et al., 2022), RGB camera fusion LIDAR (HE et al., 2022), etc., but these methods still have many problems, such as complex fusion algorithms, sparse point clouds, high cost, and complex calibration.

In recent years, with the development of deep learning, the information contained in images has been further mined, in addition to models such as target detection and instance segmentation, some scholars have proposed a monocular depth prediction model, which predicts the depth (distance relative to the camera) at each pixel position from a single image (Abuolaim and Brown, 2020), even though the training process uses sparse depth maps, the trained model is capable of outputting dense depth maps. In combination with the camera’s intrinsics, depth can also be converted into a 3D point cloud in the camera’s coordinate system (Fu et al., 2021). In a recent study, the state-of-the-art model had only 3.9% absolute relative error in predicted depth versus label on the KITTI dataset (Hu et al., 2024), demonstrating its potential for autonomous driving, robotics, 3D reconstruction, and more. In recent years, depth prediction models have begun to be applied in the field of agriculture. Zhao et al. (2022) used the P3ES-Net depth prediction model to reconstruct a 3D point cloud of a plant from a single image and measured the phenotypic parameters of the plant from the point cloud. Cui et al. (2022) used the MonoDA model to achieve monocular depth prediction in a vineyard environment, with an absolute relative error of 13.4%. Coll-Ribes et al. (2023) improved the accuracy of grapes image instance segmentation by fusing depth and RGB information, where the depth is predicted by a model, improving the F1 value from 0.882 to 0.924 compared to using only RGB images. Shu et al. (2021) built a field SLAM system using a monocular depth prediction model, which allowed the system to get rid of the LIDAR and stereo cameras and can be easily deployed on existing equipment. Although there has been an influx of research on the application of depth prediction models in agriculture, more application scenarios still need to be explored, such as monocular visual navigation.

In a recent study, we used a deep learning model to achieve instance segmentation of ratoon rice rolled stubble rows (Li et al., 2023), but in order to achieve automatic navigation, it is also necessary to locate the position of the stubble rows. In this paper, machine vision is used to realize monocular vision-based 3D spatial localization of rolled stubble rows of ratoon rice, unlike the multi-sensor fusion approach, this study adopts a deep learning model fusion-based method, where one model is used for recognition and the other is used for localization, in which the recognition model is an instance segmentation model, which is one of our previous research results (Li et al., 2023), and the localization model is a depth prediction model. We investigated the depth prediction performance of the depth prediction model under the ratoon rice field scene, and finally fused the outputs of the instance segmentation model and the depth prediction model to obtain the spatial location of the navigation line and 3D point cloud of ratoon rice rolled stubble rows.

The main structure of this paper is as follows: in Section 2, we first introduced the model fusion method, and then described the structure and training method of the depth prediction model used in this paper. In Section 3, we trained the depth prediction model, tested the model performance on a validation set, and then obtained the law of the influence of the change of the focal length of the input image on the depth value predicted by the model, according to which we proposed two optimization methods to improve the performance of the model migrating to a monocular camera. Finally, we conducted model fusion experiments.

## 2 Materials and methods

### 2.1 Method of recognizing and locating the rolled stubble rows

**Figure 1:**
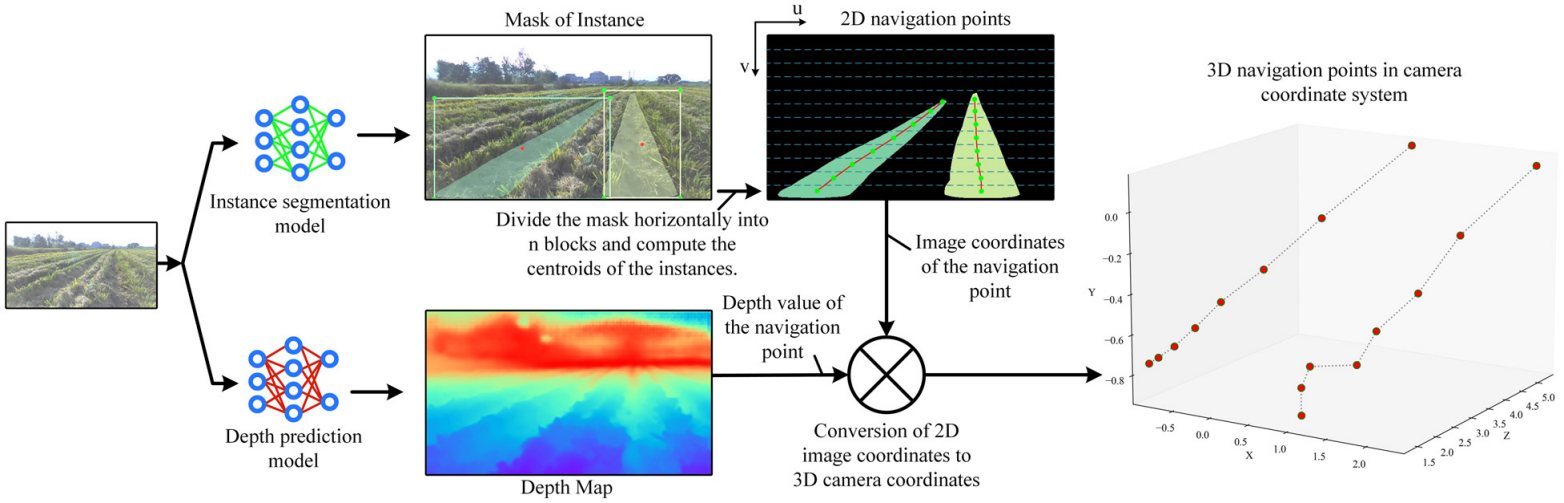
Flowchart of the navigation line localization method based on model fusion. Each color of mask represents a row of rolled rice stubble. The image coordinate system (u, v) is in pixel and the camera coordinate system (X, Y, Z) is in m. The size of the depth map is equal to that of the input image, and the valid depth value (greater than 0) on each pixel indicates the vertical distance of the object from the camera, i.e., the value of the Z axis in the camera coordinate system.


Flowchart for model fusion is shown in **Figure 1**, where an image is input into the instance segmentation model and the monocular depth prediction model to output the instance mask and the depth map, respectively. Then, the instance mask is horizontally divided into n blocks, and the average horizontal and vertical coordinates of each instance mask in each block are calculated in the image coordinate system, which are used as the coordinates of the navigation points, and the navigation lines are formed by connecting the navigation points in the instances. According to the image coordinates of the navigation point, corresponding depth value can be obtained in the depth map. Then, the planar 2D image is converted to a 3D point in the camera coordinate system according to a conversion equation between image coordinates and camera coordinates in the camera imaging principle:

(1) $$Z[\begin{array}{c} u\\ v\\ 1 \end{array}]=[\begin{array}{ccc} f_{x} & 0 & C_{x}\\ 0 & f_{y} & C_{y}\\ 0 & 0 & 1 \end{array}][\begin{array}{c} X\\ Y\\ Z \end{array}]$$

Z equals depth, u, v are image coordinates, and X, Y, Z are 3D coordinates in the camera coordinate system, $f_{x}$ and $f_{y}$ , $C_{x}$ and $C_{y}$ are intrinsic parameters that represent the pixel-represented focal length in x and y directions, the x and y coordinates of the optical center in the image coordinate system, respectively.

### 2.2 Depth dataset

The dataset is collected using two depth cameras, one for training and validation, and the other for testing the generalization ability of the trained model. This is critical in real-world deployments, as the trained models will be migrated to the monocular camera for deployment.

#### 2.2.1 Data collection in the field

**Figure 2:**
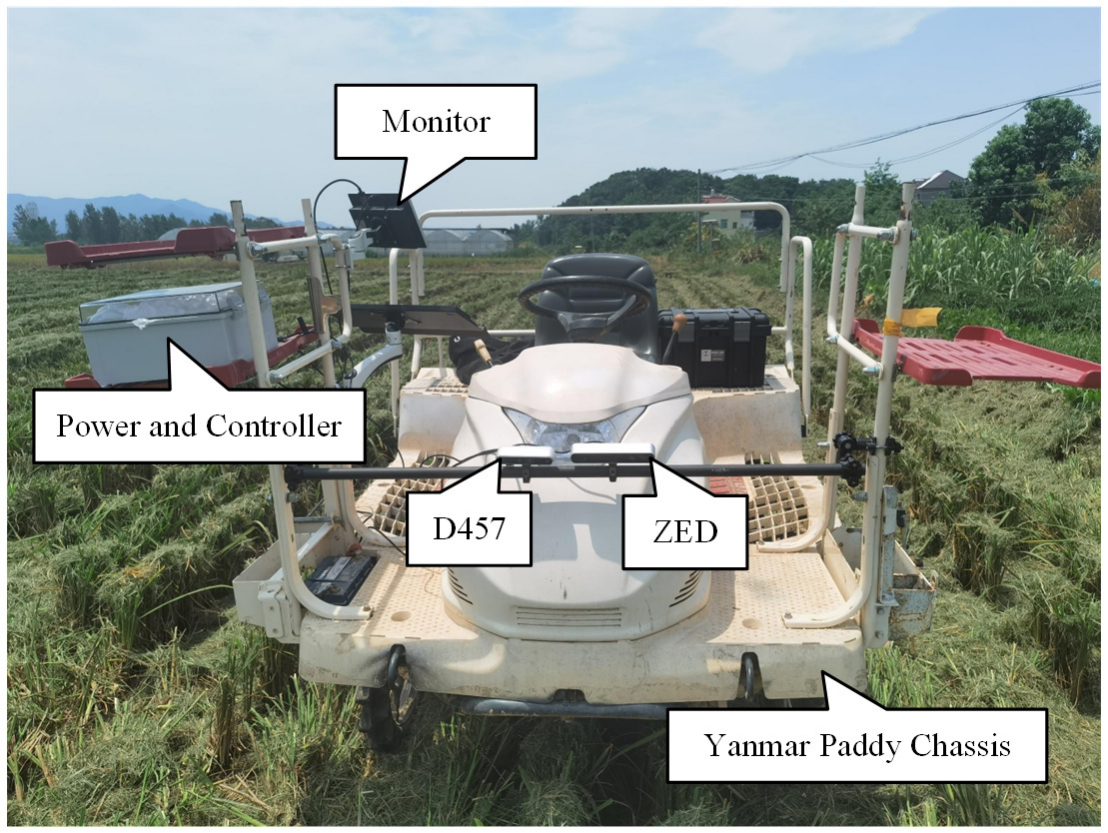
Dataset collection scene and collection equipment. The equipment consists of a power supply and controller, a monitor, two depth cameras, and a Yanmar Paddy Chassis. The controller is an AGX Xavier Orin. The images taken by the cameras are transmitted to the monitor in real time. Two depth cameras mounted side by side with lenses tilted to look at the ground.

The dataset was collected in Cailing Town, Duchang County, Jiujiang City, Jiangxi Province, China, in the mornings of August 10 and 11 2024, under sunny weather. The collection environment was a paddy field after the first harvest of ratoon rice, and the collection environment and equipment is shown in **Figure 2**, two depth cameras were used to collect data, the Intel RealSense D457 (D457) and Stereolabs ZED (ZED), the resolution of the images collected by the D457 is 1280*720, and that of the images collected by the ZED is 640*360, their models are shown in **Figure 3 of the Supplementary Material**, and setup information is given in **Table 2 of the Supplementary Material**.

As shown in **Figure 2**, Two depth cameras were mounted in front of the paddy field vehicle, and the vehicle was manually driven through the field, with the two depth cameras automatically collecting data every 0.2 seconds. As the environments in the centre of the field were very similar and the environments at the edges of the field varied considerably, the vehicle was driven around the boundaries most of the time when collecting data on a field in order to increase the diversity of the data.

#### 2.2.2 Dataset production

**Figure 3:**
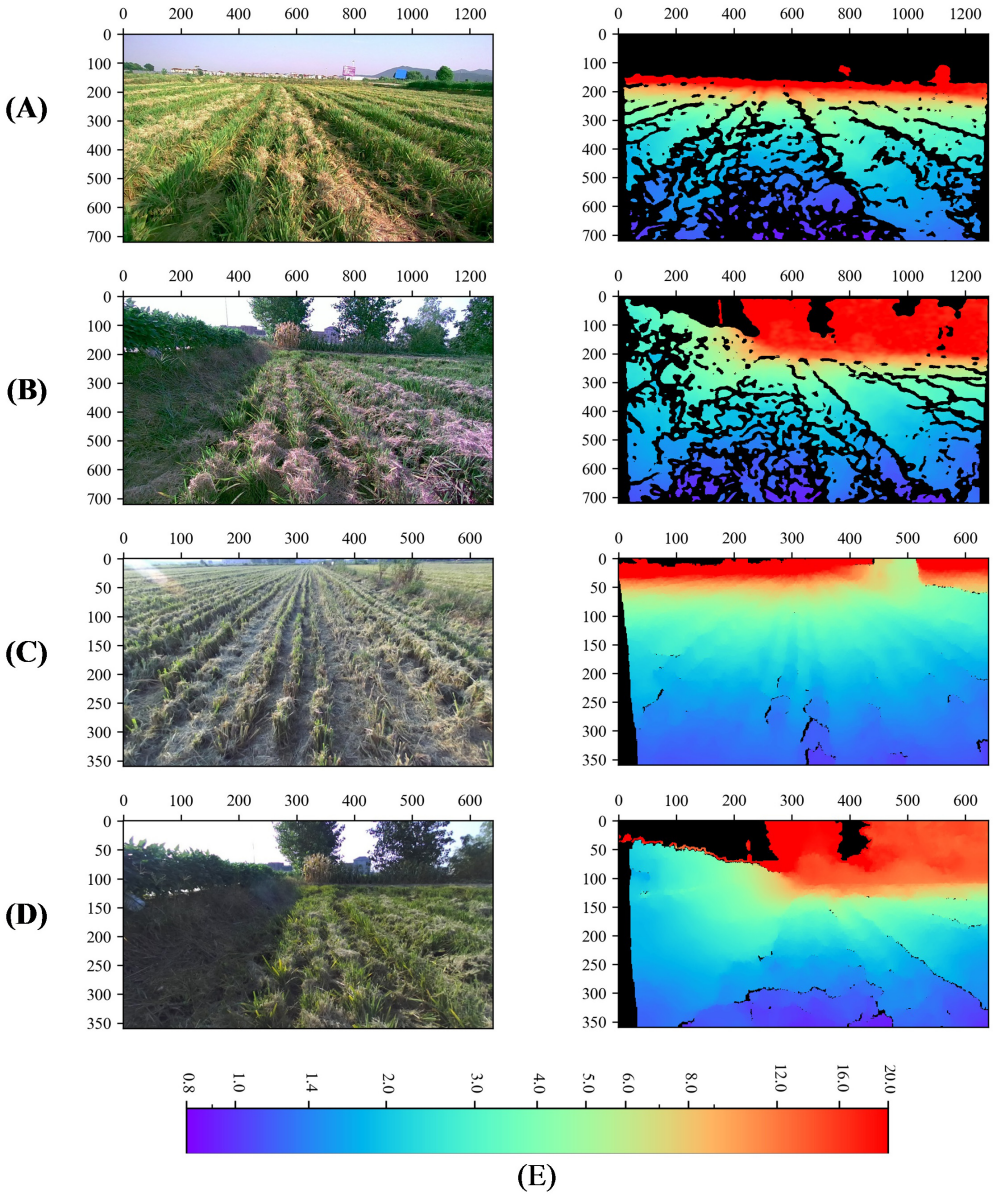
Dataset visualization. **(A, B)** denote general, obstacle type data captured by the D457 camera, **(C, D)** denote general, obstacle type data captured by the ZED camera, **(E)** Color bar for depth map, m. The first column is the rgb image and the second column is the depth map. The black part of the depth map indicates that the depth information is null here. The unit of the color bar is m.

In order to improve the reading and writing efficiency of the depth data, the png image format was adopt to store the depth data, the original data collected were of floating type with the unit of m which becomes mm after being multiplied by 1000, and the data type was converted to unsigned 16-bit integer and saved, which can retain the precision of three decimals. 33706 sets of data were collected by the two depth cameras, D457 and ZED, each set of data contains a left eye RGB image and a depth map, 19808 sets collected by the D457 depth camera, and 13898 sets collected by the ZED depth camera. All data can be categorized into general and obstacle according to the scene, several sets of data are shown in **Figure 3**, where the black part is the missing depth value. The depth values were saved in the range of 0-20 meters.

The collected data are divided into training, validation and test sets, where the training set contains 16,808 sets of data from the D457, the other 2,000 sets of data from the D457 are the validation set, and 2000 images captured by ZED were randomly selected as test set to test the performance of the model migrated to the monocular camera.

#### 2.2.3 Dataset augmentation

Data augmentation has proved to be a robust technique for solving a variety of challenging deep learning tasks, including image classification, natural language understanding, speech recognition, and semi-supervised learning (Gong et al., 2021). The main method (DNNs) to improve the generalization ability of deep neural networks is data augmentation by expanding the training set through data transformation (Dabouei et al., 2021). In this study, in order to reduce or even eliminate the effect of color during model training and to avoid the depth prediction model predicting depth based on color information, we used image enhancement techniques during model training. Before feeding the images into the model, we performed random color transformations on the images and all the transformation operations are done by Albumentations (Buslaev et al., 2020).The flow chart of transformation is shown in **Figure 1 of the Supplementary Material**, the partial data augmentation results for one image are shown in **Figure 2 of the Supplementary Material**.

### 2.3 Monocular depth prediction model

#### 2.3.1 Model structure

**Figure 4:**
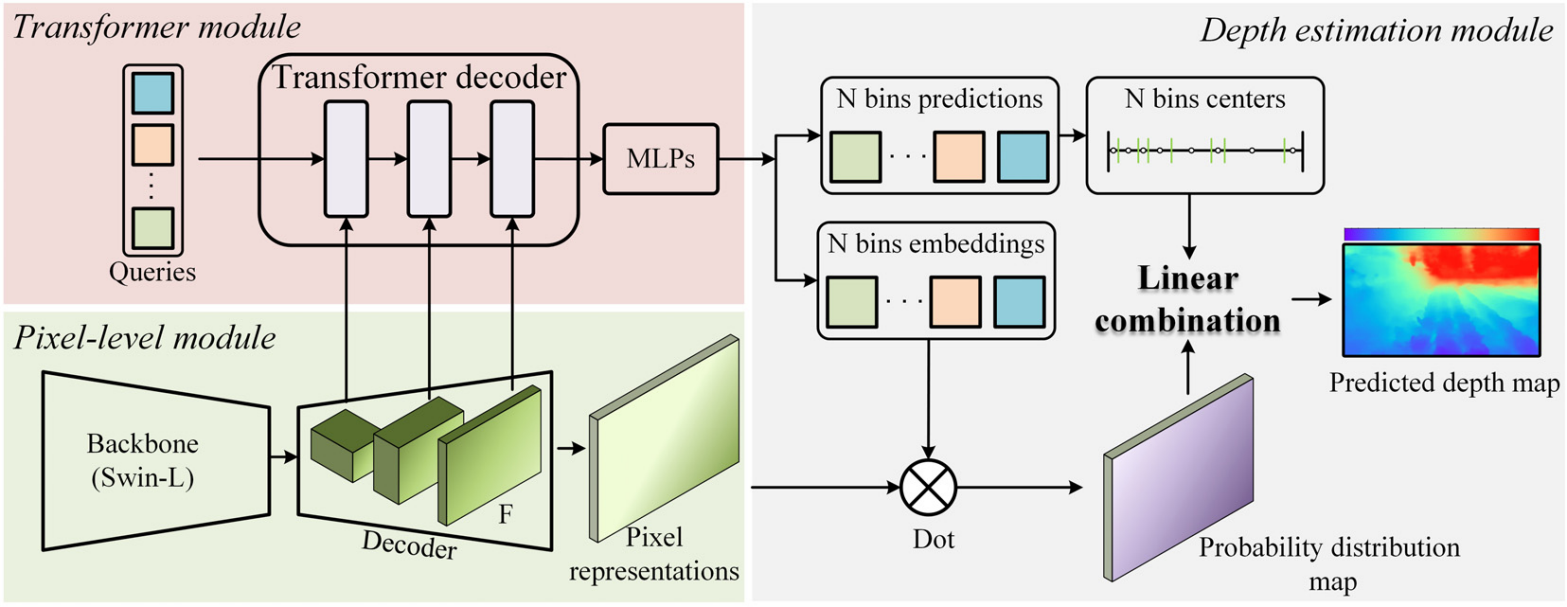
BinsFormer overview.

The depth prediction modeling framework used in this study is BinsFormer (Li et al., 2024), and its overall structure is shown in **Figure 4**, which consists of three parts: a pixel-level module, a transformer module, and a depth prediction module. An image is first fed into the backbone network (The swin-L (Liu et al., 2021) was used) in the pixel-level decoding module, which extracts features from the image and then decodes them into multiscale features F and pixel-level representations. Then, in the Transformer module, queries interact with F with the help of the attention mechanism and their outputs go into the independent MLPs. MLPs output embeddings into bins predictions and bins embeddings. Then the model predicts the probability distribution map via a dot product between pixel representations and bins embeddings in depth estimation module. The final depth estimation is calculated by a linear combination between the probability distribution map and post-processed N bins centers. The model output are absolute depths in m.

#### 2.3.2 Model training methods

**Figure 5:**
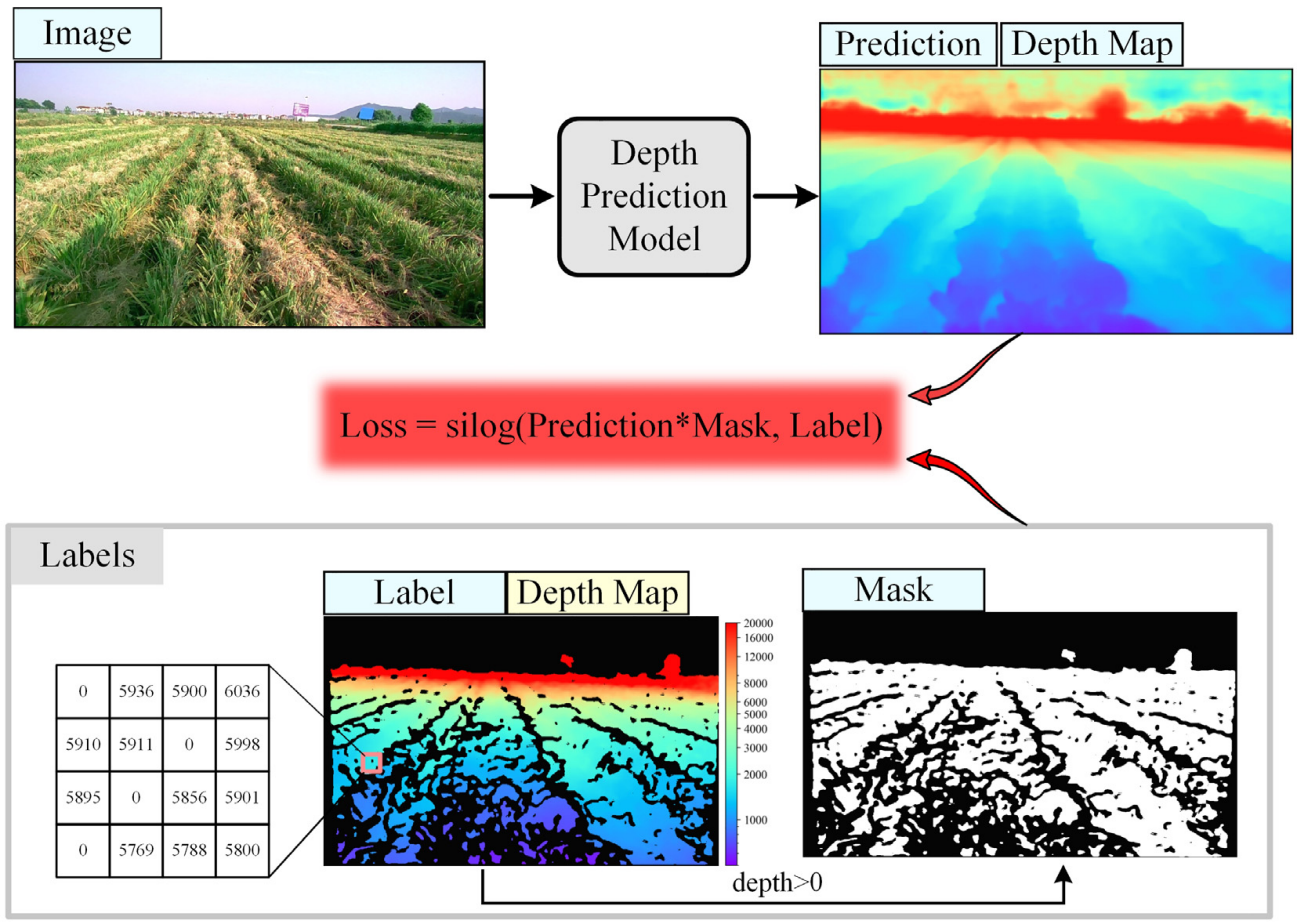
Flowchart for training of depth prediction model. The white portion of the Mask has a value of 1 and the black portion has a value of 0. 'Prediction*Mask' indicates a pixel-by-pixel multiplication of the depth in the prediction depth map and the mask.

The training process of the depth prediction model is shown in **Figure 5**. During the training process, in addition to the input RGB images for inference, labels are needed to calculate the loss, the labels include the depth map captured by the depth camera and the corresponding mask, the mask is the part of the label that is not null, the mask avoids missing part (denoted by 0) of label depth map is involved in the loss calculation. The loss function used in training is silog (Bhat et al., 2023). The data used in training is the D457 training set. The hardware used for training is mainly a Xeon (R) Platinum 8358P CPU, 10 NVIDIA GeForce 4090 24G GPUs. the training hyperparameters are shown in **Table 1**. To highlight that the model was trained on the D457 dataset, this model is uniformly referred to as ‘Model-D457’ in the following.

**Table 1:** Hyperparameters for model training.

Hyperparameters	Value
Optimizer	AdamW
Weight decay	1.00e-04
Initial learning rate	0.001
Max iters	40000
Validation interval	1000
Minibatch size	10
Learning rate scheduler	Poly
Learning rate drop power	0.5

### 2.4 Evaluation metrics

In this study, eight commonly used evaluation metrics were used to assess the accuracy performance of the depth prediction model, including absolute relative error (ABS-REL), root mean squared error (RMSE), threshold accuracy (δ1, δ2, δ3) etc., and their calculation methods are as follows.


(2) $$\text{ABS-REL}=\frac{100\%}{N}\sum |\frac{y_{pred}-y_{gt}}{y_{gt}}|$$

(3) $$\text{RMSE}=\sqrt{\frac{1}{N}\sum {\left(y_{pred}-y_{gt}\right)}^{2}}$$

(4) $$\text{Silog}=\frac{1}{N}\sum_{i}^{n}d_{i}^{2}+\frac{1}{N^{2}}{\left(\sum_{i}^{N}d_{i}\right)}^{2},\;d=log(y_{pred})-log(y_{gt})\;\;$$

(5) $$\;\delta \text{n}(\%)=\frac{100}{N}\sum_{i}^{N}max(\alpha_{i},\alpha_{i}^{*})<1.25^{n},\;\;\;\alpha =\frac{y_{gt}}{y_{pred}}\;\alpha^{*}=\frac{y_{pred}}{y_{gt}},\;\;where\;n=1,2,3$$

(6) $$\text{RMSElog}=\sqrt{\frac{1}{N}\sum {\left(log(y_{pred})-log(y_{gt})\right)}^{2}}$$

(7) $$\text{SQ-REL}=\frac{1}{N}\sum {\left(\frac{y_{pred}-y_{gt}}{y_{gt}}\right)}^{2}$$

where $y_{pred}$ is the model prediction at the pixel position and $y_{gt}$ is the depth value (not null) captured by the depth camera at the corresponding position. N denotes the total number of $y_{gt}$ in the depth map. All are smaller and better except δn which is larger and better.

## 3 Results and analysis

In this chapter, the depth prediction model was trained and its performance was tested on the validation set, then we obtained the law of the influence of focal length through the model prediction results, based on which two optimizations were proposed to improve the performance of the model migrated to a monocular camera (ZED test set). Once the model was ready, model fusion experiments were carried out to obtain the 3D coordinates of the navigation points and the rolled rice stubble rows, and the CD values between them and the labels were calculated.

### 3.1 Model training process and performance on validation set

**Figure 6:**
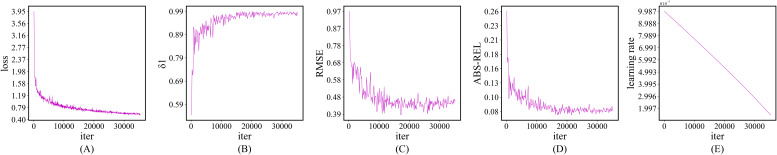
Change curves of some key values during training. **(A)** loss change curve, **(B)** δ1 change curve on validation set, **(C)** RMSE change curve on validation set, **(D)** ABS-REL change curve on validation set, **(E)** Learning rate change curve. Their horizontal directions are all in iter.

The change curves of some important values of Model-D457 during the training process are shown in **Figure 6**. As the training proceeds, the loss decreases significantly, and the evaluation metrics δ1 rises, RMSE and ABS-REL show a decreasing trend, indicating that the depth information predicted by the model is getting closer and closer to the depth captured by the depth camera. Although the loss still decreases significantly after iteration up to 10000, the accuracy on the validation set does not improve significantly, which may be due to the learning rate being too small at this time. The best RMSE is achieved on the validation set at 17000 training iterations, and the model weights at this point are saved as the final weights.

**Figure 7:**
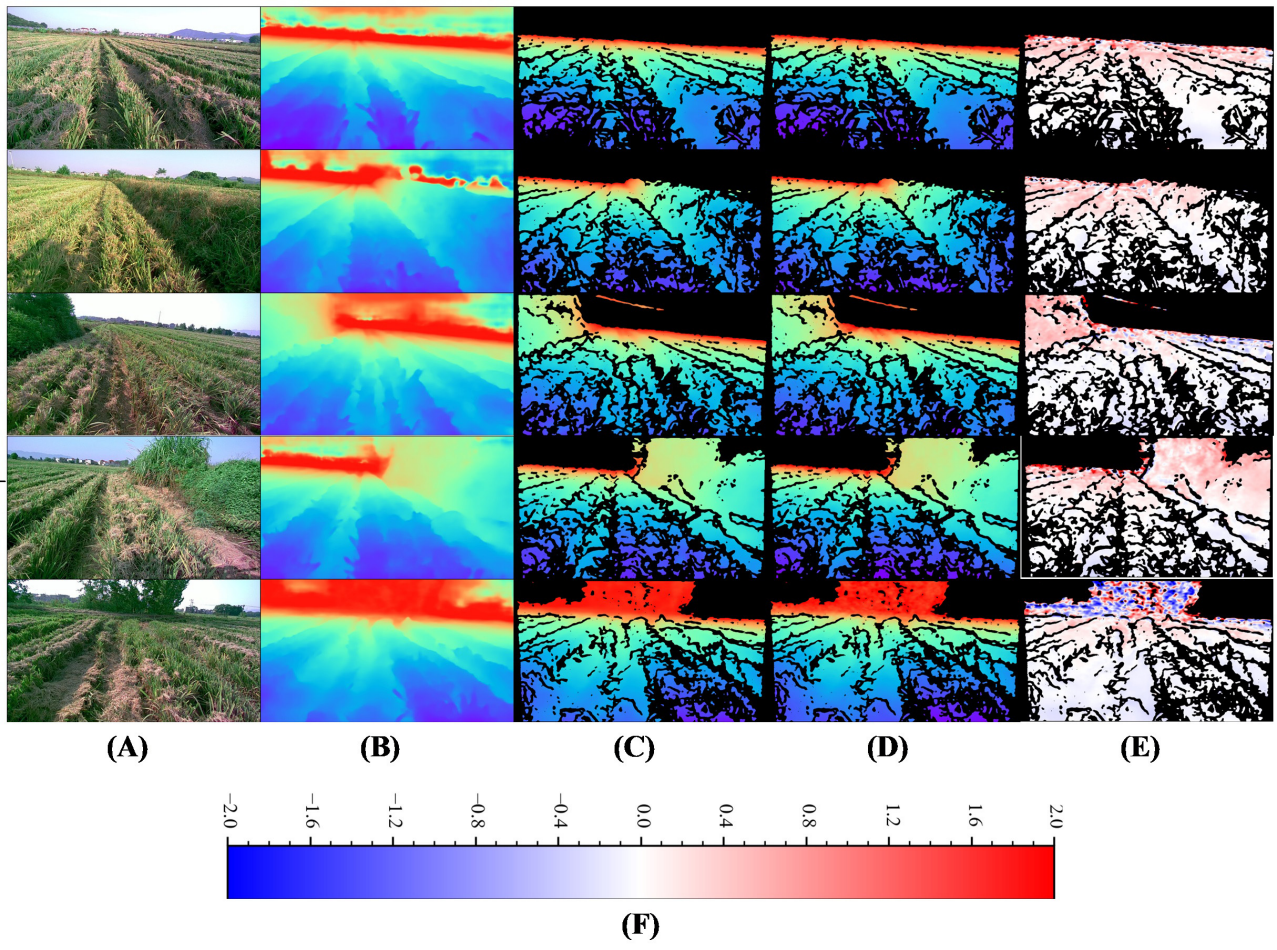
Visual comparison of Model-D457's output on the validation set with labels. **(A)** Input images, **(B)** Full predicted depth map, **(C)** Predicted depth map at label position, indicates the portion of the predicted depth map that corresponds to valid values at the label position, **(D)** Label depth map, acquired by the D457 camera, **(E)** Error map, is calculated as the valid depth value in the label depth map minus the predicted value pixel by pixel, **(F)** Color bar for error map, m. The color bar for depth map is shown in **Figure 3**.

The visualization of Model-D457’s inference results on several images of the validation set is shown in **Figure 7**. By comparing the full predicted depth maps and labels, it can be seen that despite the large number of nulls in the label depth maps used in the training process, but the predicted depth map has significant depth errors at long range due to the absence of long-distance depth label. Comparison of the label depth map and the predicted depth map at the corresponding locations shows that the depth values obtained by the model inference are very close to the label depth map in color, which is further evidenced by the error maps, where the difference between the label depths and the predicted depths is so small that it presents large areas of white color, in addition, we also find that the larger error values are concentrated at the far distance, which presents blue and red colors in error map.

In **Table 2**, the ABS-REL between the predicted and label values of the model is only 7.2%, the RMSE is only 0.383, and the other evaluation metrics also exhibit small errors. The above results show that the depth prediction value of Model-D457 is very close to the measured value of D457 depth camera, which demonstrates its strong spatial perception ability.

**Table 2:** Performance of Model-D457 on the validation set.

ABS-REL↓	RMSE↓	Silog↓	δ1↑	δ2↑	δ3↑	RMSElog↓	SQ-REL↓
7.2%	0.383	0.037	0.984	0.999	0.999	0.039	0.024

### 3.2 Effect of pixel-represented focal length on depth prediction

Pixel-represented focal length expresses the physical focal length in pixels, zooming in on an image causes pixel-represented focal length to increase, zooming out causes it to decrease, and cropping an image does not change pixel-represented focal length. Recently, a scholars have achieved the training of a depth prediction model on mixed dataset by adjusting the pixel-represented focal lengths on different publicly available datasets to be consistent (Yin et al., 2023), which demonstrated that the pixel-represented focal length of the input image has an effect on the prediction results of the model, but the exact effect is not clear, and we experimentally explore the exact effect in this section.

**Figure 8:**
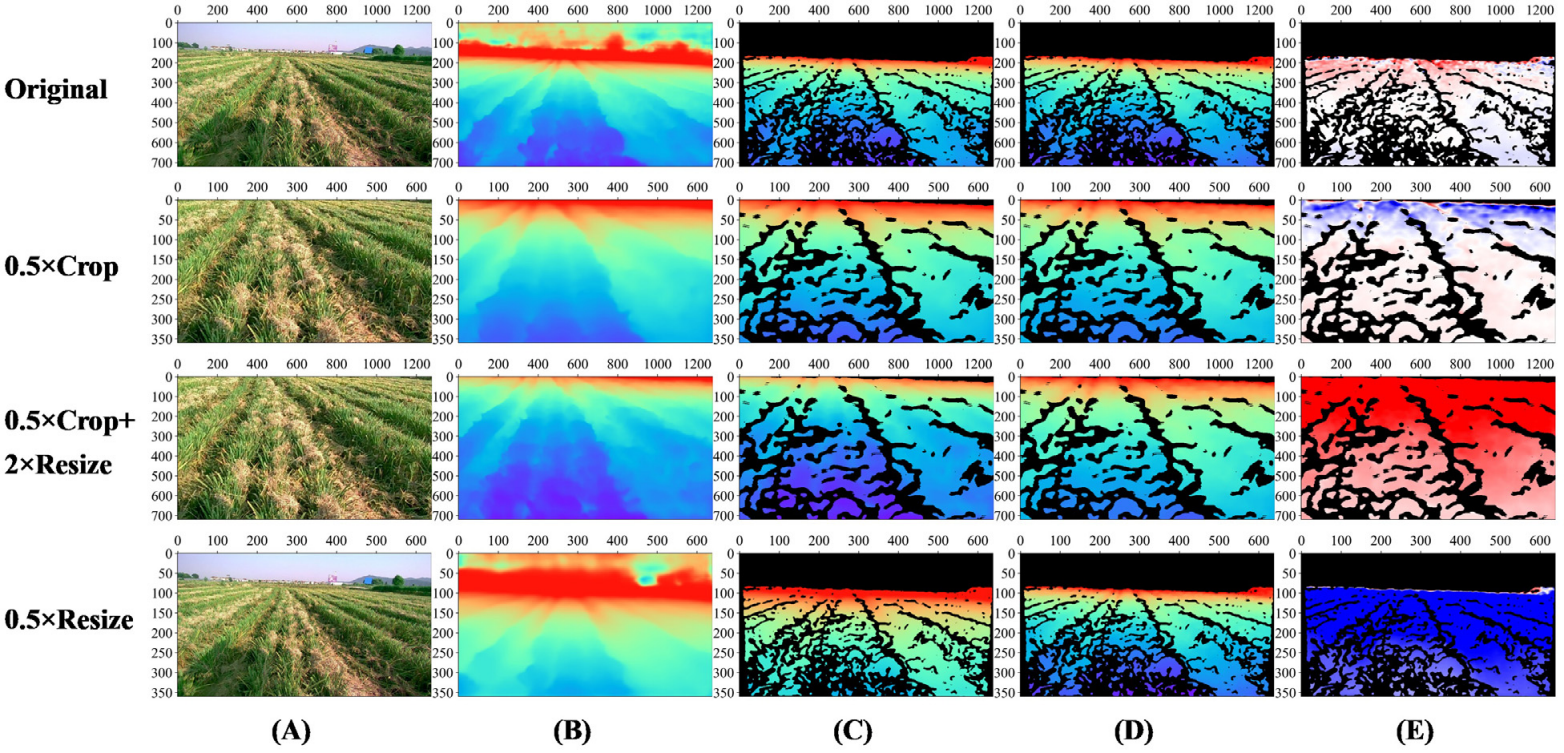
Visualization of Model-D457 output results under four transformations on one image of the validation set. Original: the result of the original image; 0.5×Crop: the result of the original image after 0.5×central cropping; 0.5×Crop+2×Resize: the result of the original image after 0.5×central cropping and 2×magnification; 0.5×Resize: the result after 0.5x reduction of the original image. **(A)** Input images, **(B)** Full predicted depth map, **(C)** Predicted depth map at label position, indicates the portion of the predicted depth map that corresponds to valid values at the label position, **(D)** Label depth map, acquired by the D457 camera, **(E)** Error map, is calculated as the valid depth value in the label depth map minus the predicted value pixel by pixel, The color bar of the error map is shown in **Figure 7**. The color bar of depth map is shown in **Figure 3**.

We carried out experiments using Model-D457 on one of the images in the validation set and obtained its outputs under four conditions, which are visualized in **Figure 8**, and the evaluation metrics are calculated in **Table 3**:

1. In the results of the original image, both the visualization results and the evaluation metrics calculations show that the predicted and label values of the model are close;
2. After the original image is centrally cropped to 360*640 from 720*1280 resolution, the predicted and label values are also close to each other, and the RMSE is slightly increased compared to that before the cropping, and the error map is reddish at distance and bluish in near area before cropping, and blueish at distance and reddish in near area after cropping, the reason for this phenomenon is still unknown;
3. After the cropped image is enlarged to 720*1280, there are obvious color differences between the predicted depth map and the label depth map, and the error map as a whole is reddish, indicating that the predicted depth value of the model is smaller than the label value as a whole, and comparing the calculation results of the evaluation metrics before and after the enlargement, the ABS-REL grows from 5% to 39.6%, the RMSE increases from 0.442 to 2.427, and the performance of other evaluation metrics also decreases significantly;
4. After the original image is reduced to 360*640, there are obvious color differences between the predicted depth map and the label depth map, and the error map as a whole is blueish, indicating that the predicted depth value of the model is greater than the label value as a whole, the performance of the evaluation metrics also decreased significantly.

**Table 3:** The performance of Model-D457 under different transformations of an image in the validation set.

	ABS-REL↓	RMSE↓	Silog↓	δ1↑	δ2↑	δ3↑	RMSElog↓	SQ-REL↓
**Original**	5.3%	0.369	0.030	0.997	0.999	0.999	0.030	0.017
**0.5×Crop**	5.0%	0.442	0.025	0.999	0.999	0.999	0.026	0.020
**0.5×Crop+2×Resize**	39.6%	2.427	0.049	0.003	0.289	0.957	0.227	0.786
**0.5×Resize**	90.6%	3.522	0.059	0.035	0.059	0.515	0.282	2.674

In conclusion, changing the pixel-represented focal length in the input image will affect the output of the Model-D457: Increasing the pixel focal length will result in smaller depths predicted by the model, and decreasing the first-estimate focal length will result in larger depths predicted by the model.

### 3.3 Adjusting inputs and outputs to improve model migration performance

**Figure 9:**
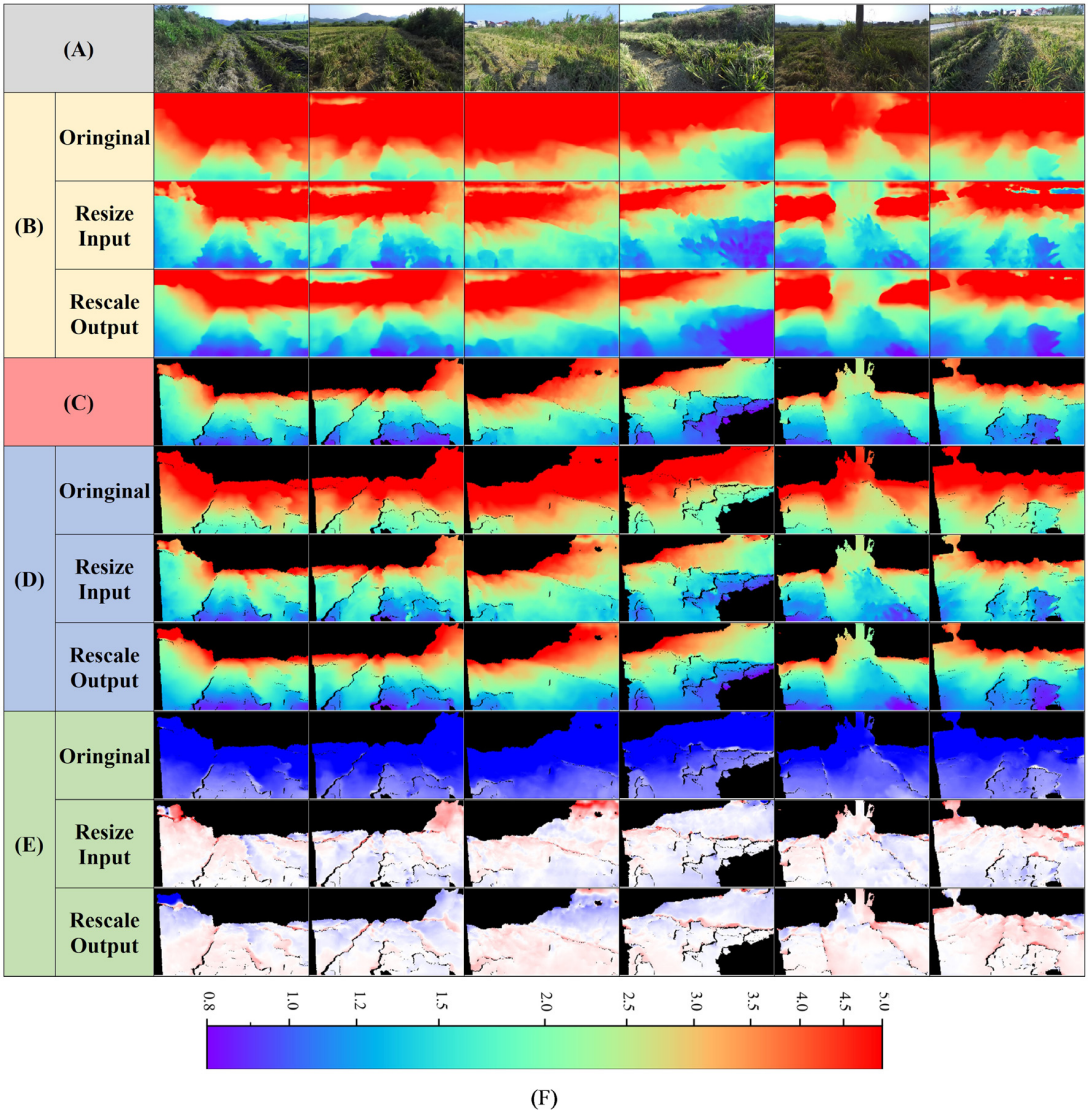
Visual comparison of Model-D457's performance on the ZED test set in three conditions. The three conditions are the original result without any manipulation, the result of resizing the input image, and the result of rescaling the output. **(A)** Input images, **(B)** Full predicted depth map, **(C)** Predicted depth map at label position, indicates the portion of the predicted depth map that corresponds to valid values at the label position, **(D)** Label depth map, acquired by the ZED camera, **(E)** Error map, is calculated as the valid depth value in the label depth map minus the predicted value pixel by pixel, **(F)** Color bar for depth map, m. The color bar for the error map is shown in **Figure 7**.

Although Model-D457 performs well on the validation set, the goal of this paper is to migrate the model to a monocular camera, which is in line with practical application scenarios, however, there is a big difference in the pixel-represented focal length between the photos taken by the D457 camera and the ZED camera, and the average evaluation metrics of all the images in the test set are shown in **Table 4**. when the original (original) ZED images are directly input into the model, it can be seen that compared to the model’s results on the D457 validation set, all the metrics are significantly decreased, the ABS-REL is more than 90%, the RMSE is more than 3.2, and the δ1 is only 0.004, indicating that the model’s performance is significantly decreased. The visualization results are shown in **Figure 9**, when the original ZED image is input, there is a significant difference in the color of the predicted depth map compared to the label at the same location, and the error value map shows blue color as a whole, indicates that the depth of prediction is greater than the depth of label.

**Table 4:** Performance of Model-D457 migrated to the ZED test set under three conditions.

	ABS-REL↓	RMSE↓	Silog↓	δ1↑	δ2↑	δ3↑	RMSElog↓	SQ-REL↓
**Original**	91.9%	3.270	0.045	0.004	0.038	0.637	0.283	2.722
**2.14×Resize**	8.8%	0.397	0.047	0.945	0.990	0.996	0.051	0.072
**Output/1.94**	8.8%	0.314	0.041	0.933	0.991	0.996	0.050	0.050

According to the experimental results in section 3.2, for the above-mentioned phenomenon that the predicted depth of the model on the test set is too large, we adopt two optimization methods namely, adjusting the input and adjusting the output to improve the performance of the model on ZED camera, and the results of average evaluation metrics are shown in **Table 4**, and results of visualization are shown in **Figure 9**:

1. Adjusting the size of the input image. When the input image of the model is enlarged to 2.14 times of the original, that is, i.e., the resolution of the input image is enlarged to 1369*770, the model performance reaches the best, and at this time, all the evaluation metrices such as ABS-REL and RMSE are significantly improved, and they are even close to the performance of the model in the validation set in **Table 2**, and the predicted depth maps of the model at this time are very close to the labels in terms of color, and the error map overall close to white, all indicating that the predicted depth values are close to the labels.
2. Adjusting the scale of the output depth values. After reducing the overall depth value by 1.94 times, the evaluation metric reaches the best, which is close to the result obtained by adjusting the input. The predicted depth maps are also very close in color compared to the labels, and the error maps are also close to white overall, indicating that the predicted depth values are close to the labels. The depth range of the labels used in the above experiment is 0-5m, because in practical use only need to obtain the distance information of the near target, plus the depth of the camera in the long distance when the accuracy is poor, this time the calculated accuracy is meaningless.

The above experimental results show that it is easy to improve the depth prediction performance of Model-D457 after migrating to the ZED camera by simply resizing the input image or scaling the model output depth overall, and its final results are even close to the model’s performance on the validation set.

### 3.4 Model fusion-based rolled rice stubble row recognition and localization experiment

**Figure 10:**
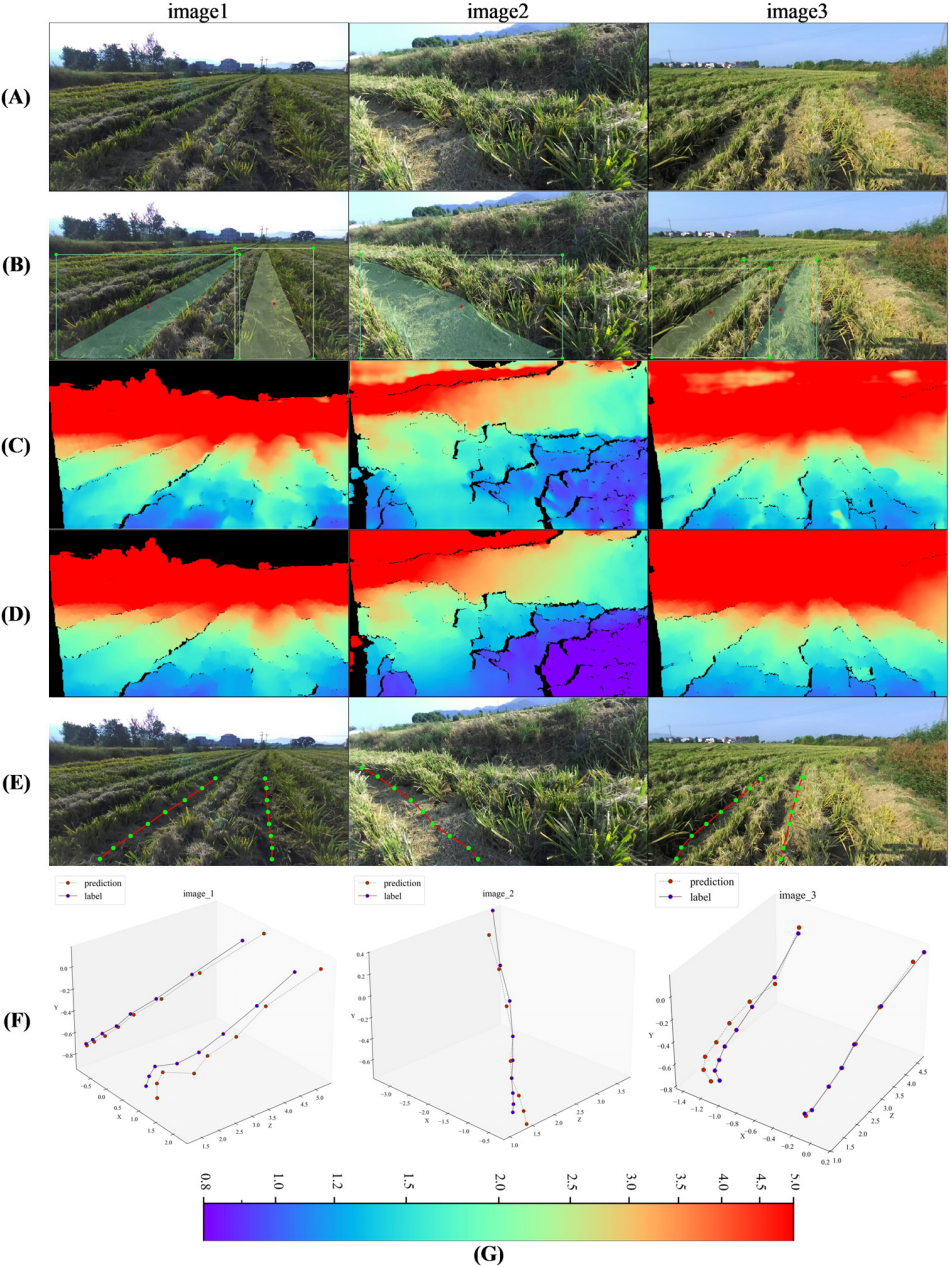
Visualization of model fusion experiment results. **(A)** Input ZED images, **(B)**Instance segmentation, the red dot in the instance mask indicates the center of the bounding box, **(C)** Predicted depth map at label position, **(D)** Label depth map, acquired by the ZED camera, **(E)** Navigation points in rgb image, only the positions of the navigation points in the mask area that are within 5m of the depth of the label are calculated, **(F)**Navigation points in camera coordinate system, including predicted and label, corresponds to the navigation point in **(E)**. **(G)** Color bar for depth map, m.

We used SMR-RS (Li et al., 2023) as the instance segmentation model and Model-D457 as the depth prediction model, and randomly selected 1000 RGB images from the ZED test set for model fusion test, of which the experimental results of three images are shown in **Figure 10**:

As shown in panels A, B and C, the images inputs to the two models output the mask of the recognized instances of the rolled rice stubble rows and the depth map, respectively, and the different color masks represent different instances, the depth maps in the figure are obtained after using the adjusted input optimization in **section 3.3**. As shown in panel E, we divided the mask horizontally of the instances into 14 rows, and calculated the average horizontal and average vertical coordinates of each instance mask in each row in the image coordinate system to obtain the image coordinates of the navigation point. Then based on these image coordinates we took out the depth value at the corresponding position from the predicted depth map and the label depth map, next, based on the equation in Section 2.1, we got the 3D coordinates of the navigation point in the camera coordinate system, **Table 1 of the Supplementary Material** (ZED-770*1369) demonstrates the intrinsics used therein. As shown in panel F, we refer to the 3D navigation points calculated from the predicted depth and label depth as ‘predicted’ and ‘label’, respectively, and we compare the label navigation points and predicted navigation points of these three images in the 3D camera coordinate system (from left to right corresponding to the rolled stubble rows in the input image), and it can be observed that their positions are very close to each other in 3D space.

CD (Chamfer Distance) (Hajdu et al., 2012) is a metric for assessing the similarity between different point clouds and is commonly used in 3D reconstruction, in this paper, the CD values between label and predicted navigation points are calculated, which is implemented using the code provided in (Christian, 2023). The average CD values of the above 1000 images obtained with (Resize Input) and without (Original) the optimization method are shown in **Table 5** (Navigation 3D Points). When the optimization method is not used, the CD value is as high as 4.70, and when the optimization method is used, the CD value is only 0.09, which is caused by the fact that the optimization method reduces the gap between the predicted depth values and the label depth values, and the gap between the 3D points obtained by depth conversion is also reduced accordingly.

**Table 5:** Average chamfer distance between predicted and label 3D points.

	Original	Resize Input
Navigation 3D Points	4.7033	0.0990
Crop Row 3D Points	1.3542	0.0174

**Figure 11:**
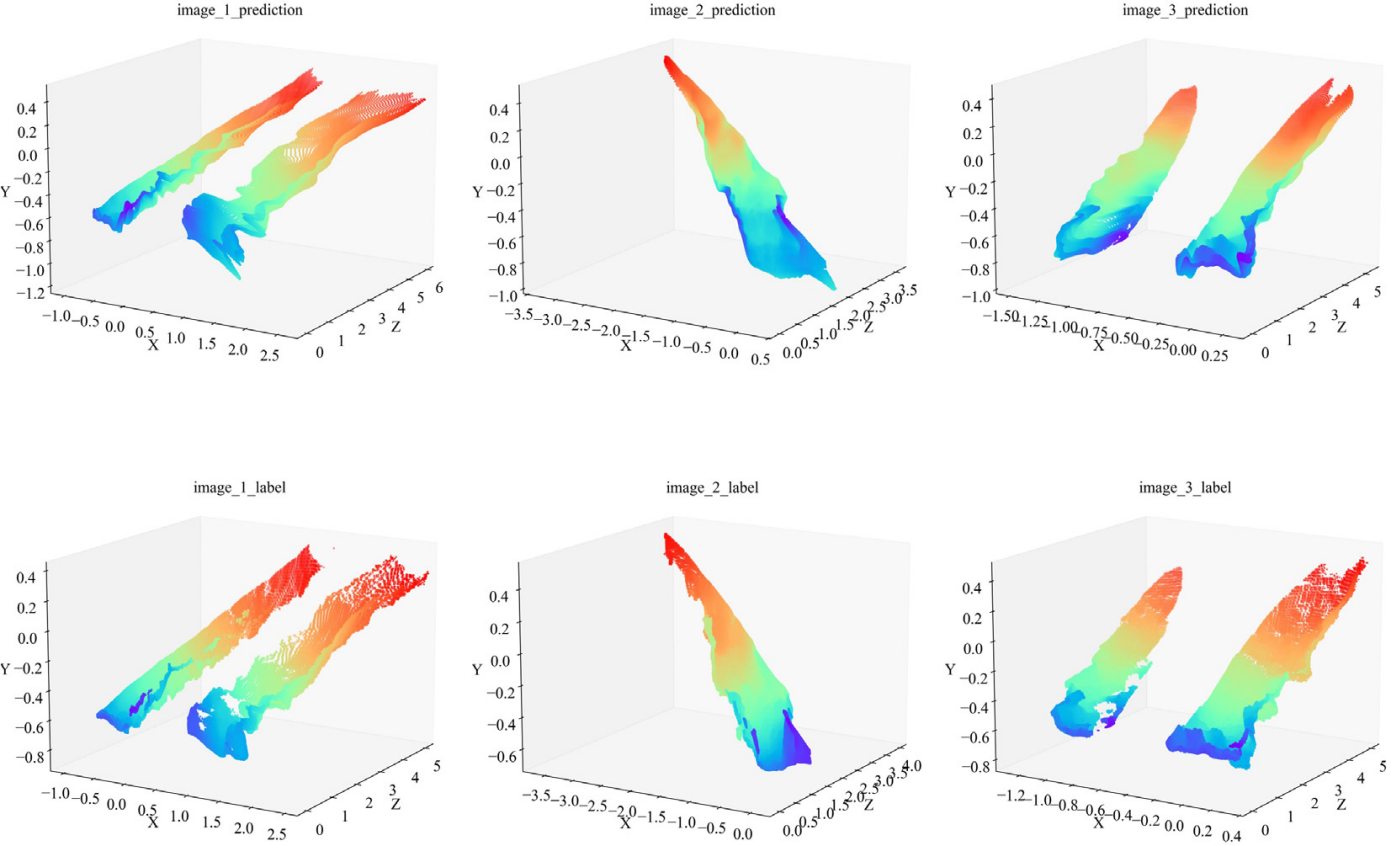
Three-dimensional point cloud of rolled rice stubble rows. The first row is the predicted point cloud and the second row is the label. The title of each plot corresponds to the image name in **Figure 10**. The color of the point cloud varies with the value of the Z coordinate and the color bar is shown in **Figure 9**.

We also obtained the label depths and corresponding predicted depths within 5 m of the instance mask location of the rolled rice stubble rows in the three images of **Figure 10**, then, used the above method, their 3D coordinates in the camera coordinate system were computed. They were plotted in **Figure 11** (the left-to-right point clouds in the single plot correspond to the left-to-right rolled rice stubble rows in the rgb image.), the first row is predicted point clouds and the second row is label, and it can be observed that they are very similar to each other. The average value of CD (Crop Row 3D Points) between the predicted and label point clouds in all 1000 test images mentioned above is calculated in **Table 5**, and the CD value is as high as 1.35 without using the optimization method. After using the optimization method in 3.4, the CD value is only 0.017.

The above results show that the model fusion-based recognition and localization method for rolled stubble rows of ratoon rice proposed in this paper can achieve high-precision spatial localization of the navigation line, and only monocular RGB images were used. In addition, the 3D point cloud of rolled stubble rows demonstrates the application of the method in 3D reconstruction.

## 4 Conclusions

In this paper, we propose a novel spatial localization method, in which we fuse the outputs of an instance segmentation model and a monocular depth prediction model and successfully achieve monocular vision-based spatial localization of ratoon rice rolled stubble rows. To realize the depth prediction, we trained Model-D457, and the ABS-REL on the validation set is only 7.2%, and the RMSE is only 0.383, demonstrating the depth prediction performance of the proximity sensor. Through experiments, we obtained the pattern of the influence of the change of the input image on the output of Model-D457: enlarging the resolution of the input image makes the model prediction result smaller, and reducing makes the model prediction result larger, accordingly, we proposed two optimization methods of adjusting the input and adjusting the output, which made the ABS-REL of the model migrated to other cameras decrease from 91.9% to 8.8%. Once the depth prediction model was ready, we conducted model fusion experiment, and the CD value between the predicted 3D coordinates of the navigation points and the labels was only 0.0990. The CD value between the predicted and label point cloud of the rolled rice stubble rows was only 0.0174. The above results show that the Model-D457 can predict depth well in ratoon rice field scene and its accuracy is even close to that of a depth sensor, applying the method of fusing the depth prediction model with the instance segmentation model we achieved localization performance close to sensor fusion (rgb fusion depth sensor), but our method is much cheaper and easier to be deployed on existing devices.

Nevertheless, our method still has some limitations. Limited by the depth measurement accuracy of the depth camera, the accuracy of the predicted depth by the depth prediction model trained in this paper at a long distance (more than 5m) needs to be improved. In addition, the inference speed of the depth prediction model is slow, and the inference time for a single image is about twice as long as that of the instance segmentation model. Based on these deficiencies, in the future, we will focus on making depth datasets with higher accuracy and at longer distances, and researching lightweight depth prediction models or end-to-end models that integrate both depth prediction and instance segmentation, and ultimately applying this method to automate rice stubble righting machines.

## Data Availability

The original contributions presented in the study are included in the article/**Supplementary Material**, further inquiries can be directed to the corresponding author/s. The code used in this paper is available at https://github.com/Yuan-rui-Li/MonoDepth.git.
