# Supplementary material for: Recognition and localization of ratoon rice rolled stubble rows based on monocular vision and model fusion
Source: Front Plant Sci. 2025 Jan 31;16:1533206. doi: 10.3389/fpls.2025.1533206 (PMC11825797; doi:10.3389/fpls.2025.1533206)
Supplement: Supplementary file 1 [file DataSheet1.docx]

Supplementary Material

## Supplementary Figures and Tables

| 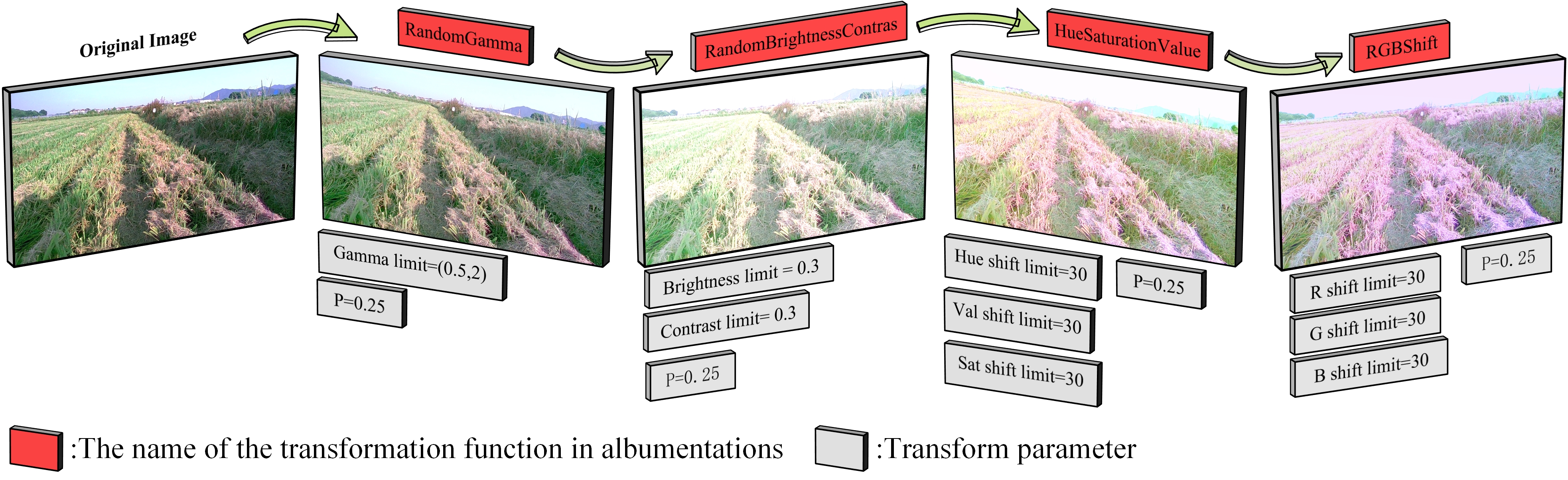 |
| --- |

Supplementary Figure 1. Flow chart for data augmentation. Consists of four steps: 1 Random gamma transformation to change the brightness and contrast of the image; 2 Random luminance and contrast transformations to further change the luminance and contrast; 3 Random hue, saturation, and color value transformations to perturb colors; 4 random RGB value offsets to further perturb colors. After the above changes, one image can theoretically be obtained as close to an infinite number of images with different colors.


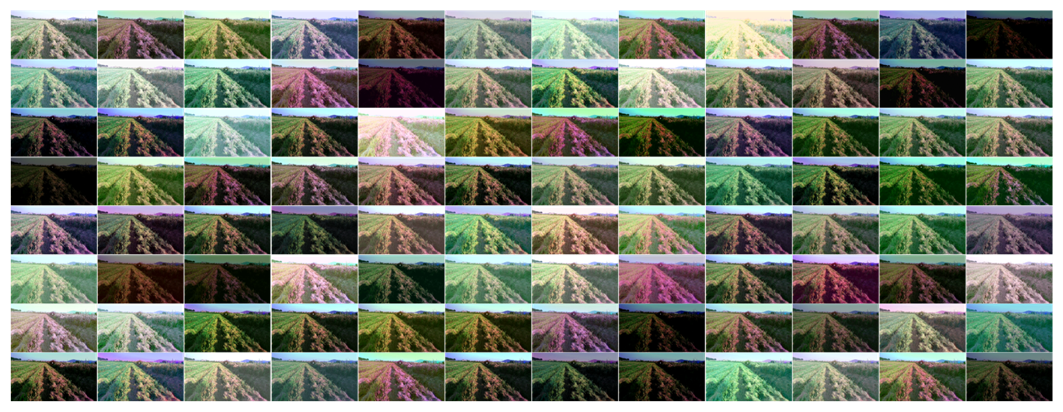


Supplementary Figure 2. 96 data augmentation results for one image.

| Supplementary Table 1. Intrinsics of the Left Eye of the ZED camera. This is the intrinsics of the ZED image at 770*1369 resolution. | | | | | |  |
| --- | --- | --- | --- | --- | --- | --- |
|  | $f_{x}$ | $f_{y}$ | | $C_{x}$ | $C_{y}$ |  |
| ZED-770*1369 | 652.18 | 652.18 | | 675.08 | 387.90 |  |
| Tip: $f_{x}$, $f_{y}$, $C_{x}$ and $C_{y}$ represent the pixel-represented focal length in the x and y directions, and the x and y coordinates of the center of the principle point in the image coordinate system. Their units are pixels. | | | | | |  |
| 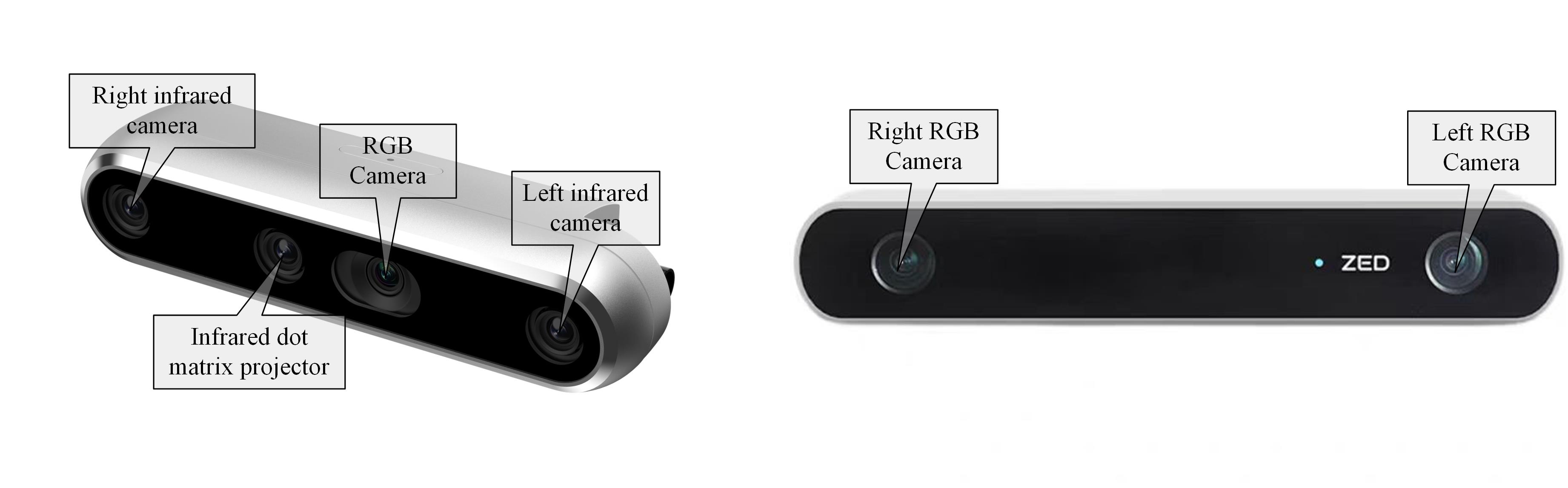 | | | | | | |
| (A) | | | (B) | | | |
| Supplementary Figure 3 Models for two depth cameras. (A)D457, (B)ZED | | | | | | |

| **Supplementary Table 2. Setup Information for Depth Camera D457 and ZED during data collection.** | | |
| --- | --- | --- |
|  | **D457** | **ZED** |
| Resolution | 720P | 360P |
| FPS | 30 | 30 |
| Depth mode | Default | Quality |
| Angle between lens plane and ground | Approx. 70°* | Approx. 70°* |
| *The pitch angle of the camera will change with the bumps of the vehicle, so the value is not fixed. | | |
